# Perspective: Evolution of Control Variables and Policies for Closed-Loop Deep Brain Stimulation for Parkinson’s Disease Using Bidirectional Deep-Brain-Computer Interfaces

**DOI:** 10.3389/fnhum.2020.00353

**Published:** 2020-08-31

**Authors:** Helen M. Bronte-Stewart, Matthew N. Petrucci, Johanna J. O’Day, Muhammad Furqan Afzal, Jordan E. Parker, Yasmine M. Kehnemouyi, Kevin B. Wilkins, Gerrit C. Orthlieb, Shannon L. Hoffman

**Affiliations:** ^1^Department of Neurology and Neurological Sciences, Stanford University School of Medicine, Stanford, CA, United States; ^2^Department of Neurosurgery, Stanford University School of Medicine, Stanford, CA, United States; ^3^Department of Bioengineering, Stanford University, Stanford, CA, United States; ^4^Graduate School of Biomedical Sciences, Icahn School of Medicine at Mount Sinai, New York, NY, United States

**Keywords:** brain-computer interface (BCI), beta oscillations, Parkinson’s disease, deep brain stimulation, subthalamic nucleus, closed-loop neurostimulation, kinematics, brain-machine interface (BMI)

## Abstract

A deep brain stimulation system capable of closed-loop neuromodulation is a type of bidirectional deep brain-computer interface (dBCI), in which neural signals are recorded, decoded, and then used as the input commands for neuromodulation at the same site in the brain. The challenge in assuring successful implementation of bidirectional dBCIs in Parkinson’s disease (PD) is to discover and decode stable, robust and reliable neural inputs that can be tracked during stimulation, and to optimize neurostimulation patterns and parameters (control policies) for motor behaviors at the brain interface, which are customized to the individual. In this perspective, we will outline the work done in our lab regarding the evolution of the discovery of neural and behavioral control variables relevant to PD, the development of a novel personalized dual-threshold control policy relevant to the individual’s therapeutic window and the application of these to investigations of closed-loop STN DBS driven by neural or kinematic inputs, using the first generation of bidirectional dBCIs.

## Introduction

Continuous deep brain stimulation (DBS) is an established therapy for cardinal motor signs in Parkinson’s disease (PD; Krack et al., 2003; Deuschl et al., 2006; Schuepbach et al., 2013; Edwards et al., 2017). Current DBS systems operate in an open-loop manner: the neurostimulator cannot sense the neural signals from the brain that interfaces with the deep brain electrode(s), and which it is modulating. It applies a continuous regular train of electrical pulses of fixed frequency, amplitude, and pulse width, which cannot automatically adjust to different symptoms, the individual’s state of activity or medication cycle. These limitations may contribute to dyskinesias and speech, mood, and cognitive impairments (Weaver et al., 2005; Deuschl et al., 2006; Williams et al., 2010). A major unmet need in neuromodulation for neuropsychiatric diseases is the development of a closed-loop neurostimulator: a bidirectional deep brain-computer interface (dBCI), in which neural signals are recorded, decoded and then used as the input commands for neuromodulation at the same site in the brain (Mussa-Ivaldi et al., 2010; Fetz, 2015).

One challenge for bidirectional dBCIs in movement disorders is the need to discover neural inputs relevant to pathological motor behaviors. Such neural inputs need to be robust, reliable, specific to the individual and their activity state, and which can be recorded during stimulation. Unlike traditional BCIs where the normal neural code is used to restore function, the neural signals available from DBS leads in PD represent pathological neural code; the desired neural activity has to be extrapolated from animals or simulations (Wichmann et al., 1994; Nini et al., 1995; He, 2014; Feingold et al., 2015). Another challenge for bidirectional dBCIs is to discover control policies (patterns and parameters of neurostimulation) that will optimize specific motor behaviors.

In this perspective, we will outline the work done in our lab regarding the evolution of the discovery of neural and behavioral control variables relevant to PD, the development of a novel personalized dual-threshold control policy relevant to the individual’s therapeutic window and the application of these to investigations of closed-loop STN DBS driven by neural or kinematic inputs, using the first generation of bidirectional dBCIs.

### Deconstructing the Resting-State Neural Code Relevant to Parkinson’s Disease

Exaggerated neuronal oscillations and synchrony in alpha and beta frequencies (8–30 Hz) have been demonstrated in the sensorimotor network during the resting state in PD, which can be termed the resting state beta oscillopathy (Bergman et al., 1994; Nini et al., 1995; Bevan et al., 2002; Levy et al., 2002; Brown, 2003; Schnitzler and Gross, 2005). Many early reports were limited to one short neural recording and it was questioned whether the beta oscillopathy was a stable feature across individuals (Priori et al., 2013). Our intra-operative recordings demonstrated that the resting state beta oscillopathy was stationary, in that it re-occurred, unchanged, over time despite intervening periods of movement or neurostimulation (Bronte-Stewart et al., 2009). In freely moving individuals with PD, the resting state beta spectral profile was conserved in different resting postures (Quinn et al., 2015). It was also similar and coherent between the STNs of an individual, although different among individuals, and was coherent with the motor cortex-STN hyper direct pathway efferent projection sites, suggesting that it is a property of the widespread sensorimotor network (de Solages et al., 2010; Whitmer et al., 2012). A computerized peak detection algorithm confirmed the presence of a resting state beta oscillopathy in 129 out of 130 STNs (Shreve et al., 2017).

Initially, it was debated whether the beta oscillopathy was an epiphenomenon or linked to Parkinson’s disease pathophysiology. Evidence suggesting that it is related to progressive pathophysiology was supported by the demonstration that it only emerged several days after inducing Parkinsonism in rodents and was not evident after acute blockade of dopamine receptors (Mallet et al., 2008), and from neural recordings in the non-human primate model of progressive Parkinsonism (Dorval et al., 2015; Muralidharan et al., 2016; Wang et al., 2017). Intra-operative bilateral STN neural recordings (112 STNs) in individuals with PD demonstrated that there was significantly greater resting-state beta band power in the more affected STN when compared to the lesser affected STN, further suggesting a relationship to disease progression (Shreve et al., 2017). As part of a longitudinal study, the resting state beta band power increased over time in the untreated STN, in two individuals with PD, who had bilateral STN DBS leads placed but who chose to have only one side activated (Trager et al., 2016).

**Figure 1:**
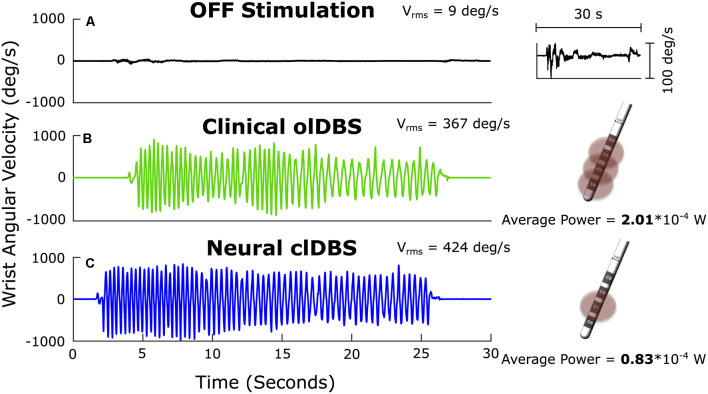
Angular velocity traces measured during repetitive wrist flexion-extension OFF deep brain stimulation (DBS; **A**); the insert on the right at higher magnitude demonstrates the severe progressive bradykinesia, ON open-loop STN DBS (olDBS; **B**) and after 60 min of closed-loop STN DBS (clDBS) **(C)**. Schematic of the DBS lead demonstrates the use of a triple monopole during olDBS and a single monopole during clDBS. V_rms_: the root mean square angular velocity averaged over the trial.

The demonstration that the STN beta oscillopathy was attenuated by therapeutic doses of dopaminergic medication and intensities of STN DBS established it as a biomarker of the Parkinsonian state (Brown et al., 2001; Levy et al., 2002; Priori et al., 2004; Kühn et al., 2006; Wingeier et al., 2006; Bronte-Stewart et al., 2009; Giannicola et al., 2010; Eusebio et al., 2011; Whitmer et al., 2012; Quinn et al., 2015). This resulted in the use of the resting state STN beta oscillopathy as a relevant neural input for adaptive DBS using a single threshold control policy, externalized leads, and a customized external neurostimulator in the acute, peri-operative state (Little et al., 2013). Consequently, the control policy that was embedded in the first generation of fully implanted bidirectional dBCIs (Activa™ PC+S-NexusE, Medtronic PLC, Minneapolis, MN, USA), was a single linear discriminator, corresponding to a single threshold of beta power. Using this we demonstrated that 60 min of closed-loop STN DBS was superior to clinical open-loop DBS for progressive bradykinesia (Figures 1B,C).

Bradykinesia (V_rms_) improved dramatically ON compared to OFF DBS (Figures 1A,B), and improved further on closed-loop DBS (Figure 1C). Progressive bradykinesia, or the waxing-waning and decreasing amplitude and speed of movement over time, was measured as the coefficient of variation of angular velocity (CV_vel_). CV_vel_ was lower on closed-loop compared to open-loop DBS (12% compared to 24%, respectively) demonstrating the superiority of closed-loop DBS. Both forms of DBS improved progressive bradykinesia compared to OFF DBS (CV_vel_ = 121%). There was a 63% reduction in the total electrical energy delivered during closed-loop DBS using a single active electrode compared to the optimized clinical DBS settings, which used a triple monopole.

### Decoding Neural Activity During Incremental Neuromodulation for Bradykinesia Led to the Development of the Dual-Threshold Control Policy Algorithm for Bidirectional dBCIs

Initially, it was difficult to discern between the effect of intensity and the effect of duration of STN DBS on the attenuation of beta band power, as there is a cumulative effect of longer periods of DBS on beta band power attenuation (Bronte-Stewart et al., 2009; Eusebio et al., 2011). We demonstrated that resting-state beta band power was attenuated in a dose-dependent manner during randomized epochs of different intensities of STN DBS (Whitmer et al., 2012).

A challenge for closed-loop DBS is to maintain a therapeutic effect while varying stimulation in real-time. Different control policies may have different goals and time scales. For instance, control policies that adjust DBS in real-time based on the appearance of symptoms [e.g., tremor or freezing of gait (FOG)] or based on rapid fluctuations of beta band power require a faster time scale (Malekmohammadi et al., 2016; Tinkhauser et al., 2017a; O’Day et al., 2020a; Petrucci et al., 2020a) compared to a control policy that adjusts DBS amplitude based on longer changes in beta band power due to fluctuating dopaminergic medication levels or the sleep-wake cycle (Arlotti et al., 2018; Velisar et al., 2019; Gilron et al., 2020). The control policies discussed in the next sections focused on time scales on the order of milliseconds. Initial single threshold control policies allowed for an “on-off” switch of neurostimulation based on the resting state beta power (Little et al., 2013); however, complete attenuation of STN beta power and/or decreasing DBS intensity toward completely off may not be optimal for motor performance (Blumenfeld et al., 2017). We developed a novel, customized, dual-threshold control policy based on the inverse relationship between DBS intensity and beta band power, and the direct relationship between DBS intensity and the improvement of bradykinesia off medication (Velisar et al., 2019). The effect of increasing DBS intensity on bradykinesia identified a minimum DBS intensity (V_min_) that resulted in the minimally acceptable improvement in bradykinesia in each individual. The beta power measured at V_min_ was chosen as the upper beta power threshold. The lower beta threshold was the beta power halfway between that corresponding to V_min_ and that corresponding to V_max_. V_max_ represented the DBS intensity above which adverse effects occurred for that individual. This resulted in a customized dual threshold policy that established a therapeutic window of DBS intensity where improvement in bradykinesia was acceptable. The policy instructed the dBCI to increase intensity when beta power exceeded the upper threshold, to stay constant when beta power remained between the dual thresholds and to decrease when beta power fell below the lower threshold. We implemented the customized dual threshold control policy and reported successful closed-loop STN DBS for bradykinesia and tremor in PD using a chronically implanted bidirectional dBCI [Activa™ PC+S-NexusD3, Medtronic PLC, Minneapolis, MN, USA (Velisar et al., 2019)]. Closed-loop DBS resulted in ~57% less total energy delivered compared to open loop DBS. We have recently demonstrated the superiority of closed loop STN DBS over clinical DBS for FOG in an individual; this experiment used beta power inputs and the customized dual threshold policy based on titrations of DBS intensity and measures of gait impairment and FOG (Petrucci et al., 2020b).

The temporal dynamics of beta band power (termed beta bursts) have been associated with clinical assessments of disease severity and with kinematic measures of bradykinesia, gait impairment, and FOG (Tinkhauser et al., 2017b; Anidi et al., 2018). There is a similar dose dependency between DBS intensity and beta burst duration both during rest and movement: increasing intensities of STN DBS were associated with shorter mean beta band durations and improved bradykinesia (Anderson et al., 2020). This dose-dependency of beta bursts suggests that a similar dual-threshold control policy in bidirectional DBCIs that monitors prolonged beta burst durations, as opposed to beta power specifically, could keep the DBS intensity within a therapeutic window.

### Optimization of Control Variables and Policies in Bidirectional dBCIs in Freely Moving Activity States

The ultimate goal of closed-loop DBS using a bidirectional dBCI is that neurostimulation will seamlessly adjust its parameters specific to the individual, their activity state, and their medication cycle (Arlotti et al., 2018). The ability to record synchronized neural and kinematic signals in freely moving individuals with PD using the implanted, sensing dBCI (Activa™ PC+S, Medtronic PLC), led to the discovery of neural and kinematic signals that corresponded to abnormal movements such as bradykinesia, gait impairment, and FOG. These recordings have demonstrated that STN beta band power can be tracked during ongoing movement in PD, that the peak frequency of the beta band did not change among rest, or finger, limb and axial movements, and that there was a subject-specific band of elevated beta power that was conserved throughout a variety of gait tasks (Blumenfeld et al., 2017; Anidi et al., 2018; Neuville et al., 2020). These contributions demonstrate that control policy algorithms in closed-loop DBS will be able to track, and do not need to adjust the frequency of, the beta band neural input in freely moving people with PD.

Such synchronized recordings also revealed STN neural signatures that differentiated individuals with PD who exhibit FOG (freezers) from those who did not freeze (non-freezers) during non-freezing gait. Beta band power was lower, mean beta burst durations were longer, and there was greater beta Sample Entropy in freezers compared to non-freezers during non-freezing gait; freezers’ gait was also more arrhythmic than that of non-freezers, even during “normal” walking (Syrkin-Nikolau et al., 2017; Anidi et al., 2018). In freezers, mean beta band burst durations were longer and alpha band (8–12 Hz) Sample Entropy was higher during periods of FOG, compared to during non-freezing gait. There was no difference in burst duration between the two groups in the resting state and burst duration was not correlated with mean power.

During open loop STN DBS at both 60 Hz and 140 Hz, gait arrhythmicity and FOG improved and beta band power and burst durations decreased in freezers (Anidi et al., 2018; O’Day et al., 2020b). The normal gait rhythmicity and shorter burst durations were left unchanged during DBS in the non-freezers. This revealed a functional relevance of beta-band burst durations as neural inputs for closed-loop DBS for gait impairment and FOG using bidirectional dBCIs. Sixty Hertz DBS resulted in improved rhythmicity in both progressive limb bradykinesia and during forward-walking tasks (Blumenfeld et al., 2017; Anidi et al., 2018; O’Day et al., 2020b). A superior effect of 60 Hz to high-frequency DBS for FOG has been reported (Moreau et al., 2008; Xie et al., 2015), suggesting the need for control policies to include adjustments in neurostimulation intensity and frequency. A method of frequency-switching would allow a bidirectional dBCI to vary both intensity and frequency for optimal behavioral improvement and finer granularity of the effects of DBS.

### Using Relevant Behavioral Signals as Inputs to dBCIs in Parkinson’s Disease

Kinematic signals specific to pathological episodic motor behaviors in PD, such as tremor and FOG, may be useful inputs to drive closed-loop neuromodulation. Resting tremor is a cardinal motor feature of PD and is an ideal behavioral input for closed-loop DBS: it is easily measured using a smartwatch, may be intermittent, and is different among individuals with PD, suggesting continuous neurostimulation may not be necessary. This was confirmed in the first behaviorally driven closed-loop DBS study, where resting tremor served as the input to the dBCI (Activa™ PC+S-NexusD system, Medtronic PLC) and dual thresholds of tremor intensity defined the control policy (Malekmohammadi et al., 2016). Resting tremor was successfully attenuated and the time that the demand-based DBS system was activated varied from 11% to 99% (average of 51.5%) of the time the continuous open-loop DBS was on. This highlights the possibility for more precise therapy for individuals with tremor-dominant PD, who may benefit from a dBCI system that is not continuous. During resting tremors, underlying beta oscillations may be attenuated and neural inputs to dBCIs may not adequately control tremors (Shreve et al., 2017; Velisar et al., 2019). Enabling the capability for an additional or back up behavioral input may be an advantage for future bidirectional dBCIs.

### Neural and Kinematic Inputs Using Intensity- and Frequency-Based Control Policies to Provide Closed-Loop STN DBS for FOG in Parkinson’s Disease

**Figure 2:**
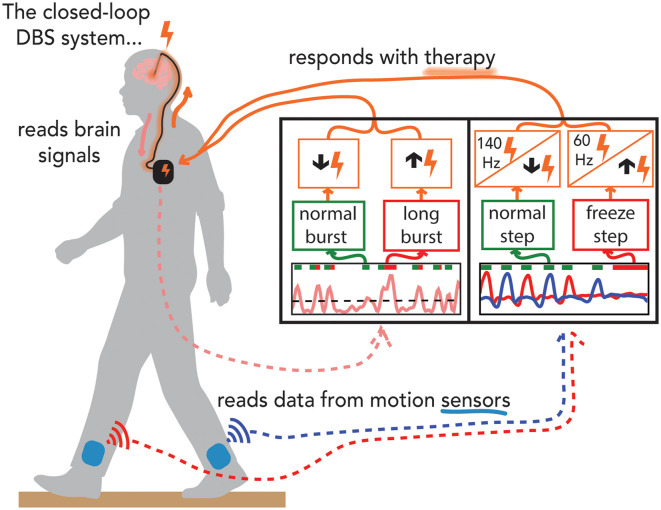
Demonstration of experiments performed on the preclinical benchtop system using the Summit application programming interface. Schematic of the fully implanted bidirectional deep brain-computer interface (dBCI) with data from the benchtop experiments. Left-hand panel: the neural input was beta band burst duration from the filtered local field potential; the single threshold control policy decided whether a neural burst was normal or long (pathological), and adapted closed-loop deep brain stimulation (clDBS) by decreasing or increasing stimulation intensity respectively. Right-hand panel: the kinematic input was the shank angular velocity streamed from wearable inertial measurement units; a dual-threshold control policy was based on whether the step was determined to be normal, uncertain, or part of freezing of gait episode and adapted clDBS by either **(I)** decreasing, not changing, or increasing stimulation intensity, OR, **(II)** by switching to 140 Hz, staying unchanged or switching to 60 Hz, respectively (right panel).

The progress in the discovery of relevant control variables and policies for closed-loop DBS in PD fueled technological advances in the capabilities of bidirectional dBCIs. This has led us to the first series of investigations into the safety, feasibility, and efficacy of both neural and kinematic closed-loop STN DBS for FOG, using relevant neural and kinematic inputs and control policies that modulate either DBS intensity or frequency, using the investigative, next-generation bidirectional dBCI, the Summit™ RC+S system (Medtronic PLC, Minneapolis, MN, USA; Figure 2; O’Day et al., 2020a; Petrucci et al., 2020a).

The Summit™ RC+S system can run both single and dual-threshold embedded algorithms. Similar to the Activa™ PC+S-Nexus-D/E systems, the Summit™ RC+S system has an Application Programming Interface (API) that allows for the development of distributed algorithms. We designed a preclinical benchtop system using the accompanying Summit API (Medtronic Inc.) that allows for external control of the RC+S neurostimulator using a PC-in-the-loop (Figure 2). The benchtop system played back previously recorded neural data and recorded output stimulation from the developed system. We used STN beta burst durations as the neural inputs and developed a novel faster time scale single threshold control policy algorithm that only increased stimulation intensity after the burst duration exceeded the inferred normal duration from simulated 1/f data, red bars in the left-hand panel in Figure 2 (Anderson et al., 2020). The benchtop system successfully adjusted stimulation in real-time in response to prolonged beta burst durations and demonstrated the feasibility of the algorithm by responding to pre-recorded STN data from an individual with PD (Petrucci et al., 2020a). We also successfully demonstrated for the first time the feasibility of kinematic closed-loop DBS for FOG using kinematic inputs relevant to impaired gait or FOG and policies that responded with adjustments of stimulation frequency or current intensity (Figure 2, right-hand panel) (O’Day et al., 2020a). This was done using real-time human subject kinematic data and kinematic data previously recorded from an individual with PD with gait impairment and FOG, allowing for real-time testing and iteration of these novel control policies using the test bench version of the Summit™ RC+S dBCI.

## Conclusion

The ability to record neural signals from DBS leads implanted in deep brain structures has made it possible to deconstruct the neural code relevant to PD and establish that the STN beta oscillopathy is a robust and reliable input for closed-loop DBS using bidirectional deep Brain-Computer Interfaces (dBCIs) in freely moving people. This led to the first demonstration of the feasibility and efficacy of closed-loop DBS for progressive bradykinesia in PD, using beta band power as the input, a single threshold control policy, and a fully embedded bidirectional dBCI. Synchronized neural and kinematic recordings during incremental DBS intensities in freely moving individuals with PD resulted in novel customized dual-threshold control policy algorithms for closed-loop DBS, where DBS intensity fluctuated within a personalized safe and therapeutic window, driven by relevant beta band power or burst duration inputs. Beta driven closed-loop DBS using the dual-threshold algorithm and an implanted dBCI was demonstrated to be safe, feasible, and efficacious for bradykinesia, tremor, and FOG. The dual threshold algorithm was also used to demonstrate the efficacy of closed-loop DBS for tremor using tremor power as the input. Neural and kinematic characterization of gait impairment and FOG in PD and during 60 Hz and 140 Hz DBS has contributed to personalized neural and kinematic inputs, and frequency and intensity-based control policies for closed-loop STN DBS therapy for FOG in PD using next-generation bidirectional dBCIs.

The advances in discovery, innovation, and collaboration have led to the next generation of fully embedded investigative bidirectional dBCIs, in which both single and dual-threshold control policy algorithms are available (Percept™, Summit™ RC+S, Medtronic PLC, Minneapolis, MN, USA). Evolution in the understanding of relevant inputs and control policies in the first generation bidirectional dBCIs for PD and epilepsy has fueled similar discoveries for treatment of other neuropsychiatric disorders (Kundu et al., 2018; Rudebeck et al., 2019; Senova et al., 2019; Mankin and Fried, 2020). Advances in the understanding of the oscillopathies and circuitopathies of neuropsychiatric diseases are developing in parallel with advances in bidirectional dBCI technology. This is contributing to a paradigm shift in therapy, which will be more precise, customized to an individual’s neural code, and will seamlessly adjust to their state of activity and medication cycle.

## Data Availability Statement

The raw data supporting the conclusions of this article will be made available by the authors, without undue reservation.

## Ethics Statement

The studies involving human participants were reviewed and approved by the Stanford University Institutional Review Board. The patients/participants provided their written informed consent to participate in this study.

## Author Contributions

HB-S, MP, JO’D, JP, YK, KW, GO and SH contributed to the conceptualization of the work and the writing and editing of the manuscript. MP, JO’D, MA, and KW contributed to the visualization of the results. MA contributed to software and data curation, as well as formal analysis.

## Conflict of Interest

Dr. HB-S serves on the Scientific Advisory Board for Medtronic PLC. The remaining authors declare that the research was conducted in the absence of any commercial or financial relationships that could be construed as a potential conflict of interest.
